# High-definition optical coherence tomography: adapted algorithmic method for pattern analysis of inflammatory skin diseases: a pilot study

**DOI:** 10.1007/s00403-012-1311-8

**Published:** 2013-01-06

**Authors:** Marc Boone, Sarah Norrenberg, Gregor Jemec, Véronique Del Marmol

**Affiliations:** 1 Department of Dermatology, Hôpital Erasme, Université Libre de Bruxelles, 128 Kardinaal Sterckxlaan, 1860 Meise, Belgium; 3 Department of Dermatology, Hôpital Erasme, Université Libre de Bruxelles, 808 Route de Lennik, 1070 Bruxelles, Belgium; 2 Department of Dermatology, Health Sciences Faculty, Roskilde Hospital, University of Copenhagen, Copenhagen, Denmark

**Keywords:** High-definition optical coherence tomography, Reflectance confocal microscopy, Inflammatory skin diseases, Ackerman’s algorithmic method of pattern recognition

## Abstract

High-definition optical coherence tomography (HD-OCT) is a non-invasive technique for morphological investigation of tissue with cellular resolution filling the imaging gap between reflectance confocal microscopy and conventional optical coherence tomography. The aim of this study is first to correlate dermatopathologic descriptors of inflammatory skin conditions with epidermal alteration to features observed by HD-OCT. Secondly, to assess the discriminative accuracy of common inflammatory reaction patterns with epidermal alteration using HD-OCT by applying Ackerman’s algorithmic method of pattern recognition. The generated HD-OCT images of 160 patients presenting an inflammatory skin disease were analyzed with respect to the following criteria: visualization of individual cells in the epidermis and dermis and morphology of dermo-epidermal junction, papillary dermis and reticular dermis. A set of morphological features corresponding to dermatopathological descriptors are obtained and the discriminative accuracy of HD-OCT of inflammatory reaction patterns could be demonstrated. These patterns are spongiotic dermatitis, psoriasiform dermatitis, interface dermatitis and ballooning dermatitis. Additional studies to test the sensitivity and specificity of the proposed algorithm for pattern analysis are essential. The other categories of Ackerman’s pattern recognition need to be evaluated. This study provides a set of morphological features generated by HD-OCT imaging very similar to those described for reflectance confocal microscopy but with the advantages not only to visualize individual cells up to a depth of 570 μm but also in both slice and en face mode. An adapted algorithmic method for pattern analysis of common inflammatory skin diseases could be proposed. This new technique appears to be a promising method for non-invasive diagnosis, evaluation and management of common inflammatory skin diseases.

## Introduction

Most dermatological diseases were originally defined morphologically, and terms such as papules and pustules are still important when diagnosing skin disease. Later dermatopathology evolved with its own special vocabulary of terms used to describe histological skin alterations, allowing dermatopathologists to classify skin diseases in great detail [21].

In dermatopathology, the initial approach is often to classify the changes broadly according to whether a process is predominantly inflammatory, predominantly proliferative/neoplastic, both inflammatory and proliferative or non-inflammatory/non-proliferative. In general, the majority of pathologic processes can be classified into one of these groups, although challenging exceptions occur, particularly when overlapping features appear [8]. Inflammatory diseases are dynamic, and knowledge of the point of time when a dermatitis is biopsied is essential to optimal microscopic evaluation.

Following the first sorting, more detailed diagnosis is then produced by identifying the specific qualitative and quantitative characteristics. Specific stains and biomarkers aid this process, but it has been suggested that overall pattern analysis is both specific and sufficient in the practical management of many diseases. Ackerman and colleagues [1] thus defined eight basic patterns of inflammatory diseases of the skin, where the pattern strongly indicates diagnosis prior to more detailed studies.

One of the challenges of skin imaging is diagnostic accuracy in comparison to the gold standard of histopathology. Because of the optical complexity of skin, which contains numerous reflective elements, two essential options exist for in vivo imaging: either sufficient optical resolution is achieved in a sufficient volume of skin to allow diagnosis, with accuracy comparable to that of histopathology, or in vivo methods have to rely on pattern recognition at a lower magnification. Combining in vivo imaging with the conceptual approach by Ackerman and colleagues may therefore be a significant step forward for skin imaging.

Reflectance confocal microscopy imaging of inflammatory skin conditions with epidermal alterations has already been studied in detail. These studies provide a set of well-described morphological criteria with obvious diagnostic impact [2–6, 14–17, 19, 20, 22, 23, 25, 26]. One limitation of this technique is that the assessment of microanatomic structures can be performed only to a depth of maximum 250 μm. A broader field of view and a greater penetration of the tissue can be achieved with OCT at the cost of resolution.

High-definition optical coherence tomography (HD-OCT) is a new non-invasive technique for morphological investigation of tissue with cellular resolution filling the imaging gap between reflectance confocal microscopy and conventional optical coherence tomography [9–11].

The aim of this study was therefore first to correlate dermatopathologic descriptors of inflammatory skin conditions with features observed by HD-OCT. Secondly, to evaluate the discriminative accuracy of common inflammatory reaction patterns with epidermal alteration using HD-OCT by applying Ackerman’s algorithmic method of pattern recognition.

## Materials and methods

### Subjects

A total of 160 fair-skinned (Fitzpatrick types II and III, 99 females and 61 males) subjects with an age range of 18–80 years (Table 1) with an inflammatory skin disease participated in this comparative study. All provided informed consent. Of these, 93 had allergic contact dermatitis (1 histologically verified and 93 positive patch tests) and 30 atopic dermatitis (fulfilling the diagnostic criteria of Hanifin and Rajka, 2 histologically verified), 9 lichen planus (histologically verified), 2 erythema multiforme (histologically verified), 3 discoid lupus erythematosus (histologically verified) and 23 chronic plaque psoriasis (3 histologically verified).

**Table 1:** Characteristics of 160 patients according different subgroups of perivascular dermatitis

Spongiotic dermatitis (*n* = 123)							
Allergic contact dermatitis (*n* = 93)						Atopic dermatitis (*n* = 30)	
Acute (patch testing)		Subacute		Chronic		Subacute & chronic	
F	M	F	M	F	M	F	M
58	18	3	2	8	4	17	13
18–69 years	18–73 years	41–74 years	19 & 66 years	23–80 years	47–79 years	18–53 years	18–59 years

Interface dermatitis (*n* = 12)				Ballooning dermatitis (*n* = 2)		Psoriasiform dermatitis (*n* = 23)	
Lichenoid		Vacuolar					
F	M	F	M	F	M	F	M
6	3	1	2	1	1	8	15
24–63 years	17–67 years	46 years	32 & 53 years	34 years	46 years	46–64 years	23–70 years

### High-definition optical coherence tomography

High-definition OCT is based on the principle of conventional OCT, with 3 μm resolution in both transversal and axial directions, to visualize individual cells (Skintell^®^, Agfa Healthcare). Moreover, the system is capable of capturing a slice image and an en face image in real time, as well as fast 3D acquisition. The tissue penetration depth goes up to 570 μm. Further technical details are discussed elsewhere [9–11].

### Intermediate (10×) magnification

The standard light microscope is characterized by an adjustable lateral resolution of 4 μm up to 0.1 μm depending on the objective and a fixed axial resolution of 5 μm. The circular field of view is 20 mm up to 0.2 mm in diameter depending on the objective used. HD-OCT is characterized by a fixed lateral and axial resolution of 3 μm. The field of view is fixed at 1.8 × 1.5 mm. The intermediate (10×) magnification of the standard light microscope (characterized by a circular field of view of 4 mm and a lateral resolution of 3 μm) corresponds with the magnification obtained with HD-OCT.

### Method

HD-OCT was done on the lesional and unaffected, clinically normal looking paralesional or contralateral skin of 160 patients with a well-established diagnosis based on history and clinical examination by a board-certified specialist of dermatology. If needed to clarify the diagnosis cultures, patch testing and any other procedures including biopsies were taken. HD-OCT imaging was done prior to any procedure.

The generated pictures were analyzed with respect to the following criteria: visualization of individual cells in the stratum corneum, stratum granulosum, stratum spinosum and morphology of dermo-epidermal junction, papillary dermis and reticular dermis.

Measurements of epidermal thickness have been performed using the measurement toolbar of the Skintell^®^ program. Each voxel of the 3-dimensional HD-OCT image is defined by a unique set of *x*, *y*, *z* coordinates. These coordinates permit to measure with accuracy the epidermal thickness.

Measurements were carried out in a climate-controlled room, at 24 °C and 40 % relative humidity.

Hematoxylin and eosin (H&E)-stained histologic vertical sections were correlated with the corresponding cross-sectional HD-OCT images but also with the en face images.

First, descriptive dermatopathological terms derived from reflectance confocal microscopy were applied to HD-OCT image (Table 2). Secondly, to evaluate relevant inflammatory reaction patterns in HD-OCT images, Ackerman’s algorithmic method of pattern recognition in the perivascular dermatitis group was applied (Table 3).

**Table 2:** High-definition optical coherence tomography descriptors

Histological terms	HD-OCT features
Spongiosis	En face: darker area relative to surrounding epithelium of stratum corneum. Intercellular spaces between keratinocytes larger than normal (Figs. 1, 2: yellow circle)
Exocytosis	En face: high reflective small dots corresponding to lymphocytes observed at the level of the stratum spinosum in single or in small aggregates (Figs. 1, 2: white arrow)
Spongiotic blisters/vesicles	Cross-sectional and en face: dark round to poly-lobulated areas could be observed (Fig. 1: yellow arrow)
Acanthosis	Cross-sectional: thickening (+10 to ±300 %) of the epidermis compared to normal [12, 13, 18, 24], mainly of the stratum spinosum. Often associated with hyperkeratosis. The epidermis can be uniformly thickened (Figs. 3, 4: green double arrow) or a disproportionate expansion of rete ridges can be observed (Figs. 6, 7)
Hyperkeratosis	En face: dark zones between entrance signal and stratum granulosum (Figs. 3, 6, 7: red arrow). Cross-sectional: the thickness of the stratum corneum was measured. A total thickness of >20 μm was measured (Figs. 3, 6, 7)
Parakeratosis	In both en face and cross-sectional: highly reflective nucleated structures in stratum corneum (Fig. 4: violet arrow)
Papillomatosis	En face: increased number and density of dermal papillary rings at the dermo-epidermal junction (Figs. 3, 4: orange-colored circle and arrow)
Hypergranulosis	In cross-sectional and en face: increase in thickness of stratum granulosum (normally 1 or 2 layers thick). This is usually seen in association with acanthosis and orthokeratotic hyperkeratosis (Figs. 6, 7: light-green arrow)
Hypogranulosis	In cross-sectional and en face: the granular layer is reduced or absent. This is always an abnormal condition and very often associated with parakeratotic hyperkeratosis (Figs. 4, 5)
Necrotic keratinocytes	In cross-sectional and en face: at the level of stratum spinosum as single units and at suprabasal layer as aggregates. They appeared as bright, polygonal structures, larger than the surrounding keratinocytes (Figs. 6, 8)
Keratotic follicular plugging	In cross-sectional and en face: plugging of the dilated openings of hair follicles by masses of keratin. A feature of a limited number of skin conditions such as discoid lupus erythematosus (Fig. 7)
Interface changes	In cross-sectional and en face: morphological alteration at the junction or interface between the epidermis and dermis. Specifically one could observe high reflective small dots corresponding to lymphocytes at the level of the junction, as singles or clusters associated with total (band like) or partial (patchy) obliteration of the papillary rings (Figs. 6, 7)
Perivascular inflammation	In cross-sectional and en face mode: high reflective small dots corresponding to lymphocytes clustered around blood vessels (Fig. 2: violet circle)
Dilated blood vessels	In cross-sectional and en face mode: prominent round or linear dark canalicular structures within papillary dermis (Fig. 3: violet arrow)

**Table 3:** Discriminative accuracy of high-definition optical coherence tomography of common inflammatory reaction patterns

	Spongiotic dermatitis	Interface dermatitis		Ballooning dermatitis	Psoriasiform dermatitis
	Ac → Chr	Vacuolar	Lichenoid		
Spongiosis	(+)++ → +	−/+	−/+	−/+	−/+
Exocytosis	(+)++ → +	+	+	+	++
Spongiotic blisters/vesicles	(+)++ → +/−	−	−	−	−
Acanthosis	− → (+)++	+++ → + (irregular)	+++ → + (irregular)	+++ → + (irregular)	+++ (regular)
Epidermal atrophy	−	+ → − (chronic)			
Papillomatosis	− → +(+)	−	−	−	++
Hyperkeratosis	− → (+)+	+	+	+	++
Parakeratosis	− → +	− (rare)	− (rare)	− (rare)	++
Hypergranulosis	−	−	−	−	−
Hypogranulosis	− → +	−	−	−	−/+
Acantholysis	−	−	−	−	−
Necrotic Keratinocyte	+/− → −	+	+	+ (ballooning)	−
Dyskeratosis	+/− → −	+	+	+	−
Honeycomb pattern preserved	−/+ → (+)+	−	−	−	++
Epidermal disarray	−	−	−	−	−
Infundibula: keratotic plug	+/−	++	−	−	−
Interface dermatitis	−	+++ (focal)	+++ (diffuse)	+++ (focal)	− (swollen, edema DP)
Dermal edema	+++ → +	++	+	+	+
Dilated bloodvessels	(+)++ → (++)+	++	+/−	++/+	++ (in dermal papilla)
Perivascular inflammation	(+)++ → (++)+	++	++	++	+++
Adnexial inflammation	−	+/−	−	−	−

## Results

### Systematic description of inflammatory changes as seen in HD-OCT (Tables 2, 3)

A set of morphological features generated by HD-OCT imaging very similar to those described for reflectance confocal microscopy are summarized in Table 3. By looking at the slice image, one can identify the exact position (stratum corneum, stratum granulosum, stratum spinosum, stratum basale, papillary dermis) of the chosen en face image. The combination of both modes permits the exact micro-anatomic localization. This particular feature is only possible with the HD-OCT. This makes it very easy to distinguish, e.g., parakeratosis from hypergranulosis.

#### Spongiotic dermatitis

**Fig. 1:**
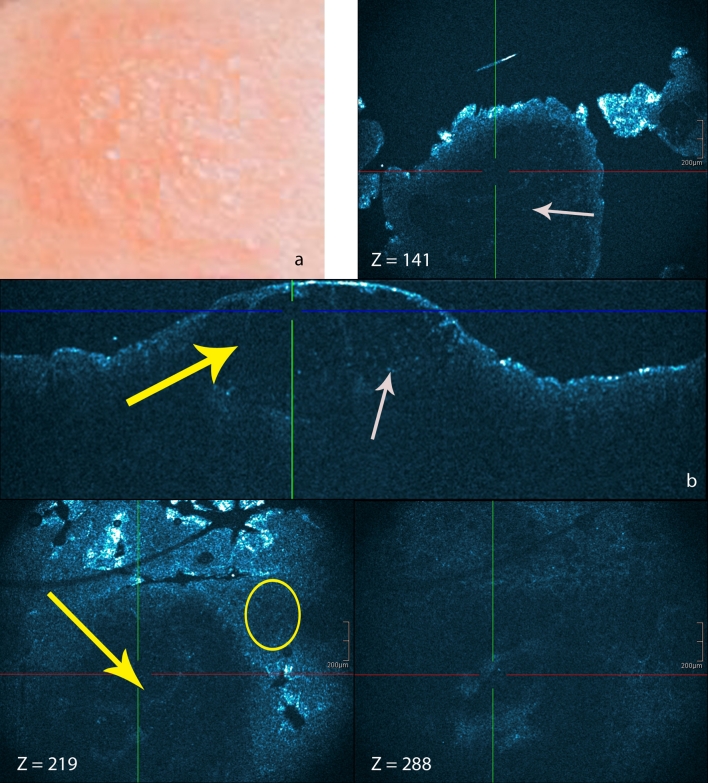
Acute allergic contact dermatitis: (**a**) patch testing graded +++ for ethylenediamine hydrochloride. HD-OCT imaging [cross-sectional (**b**) and en face with *z* values] clearly shows the presence of spongiosis or intercellular edema (*yellow circle*) that stretches apart keratinocytes and results in the formation of intraepidermal (macro)vesicles (*yellow arrows*). Exocytosis (*white arrows*) of inflammatory cells can be demonstrated. There is no hyperkeratosis. Intercellular edema decreased the reflectivity from cellular layers. *z* values indicate the depth of the en face image (in μm)

**Fig. 2:**
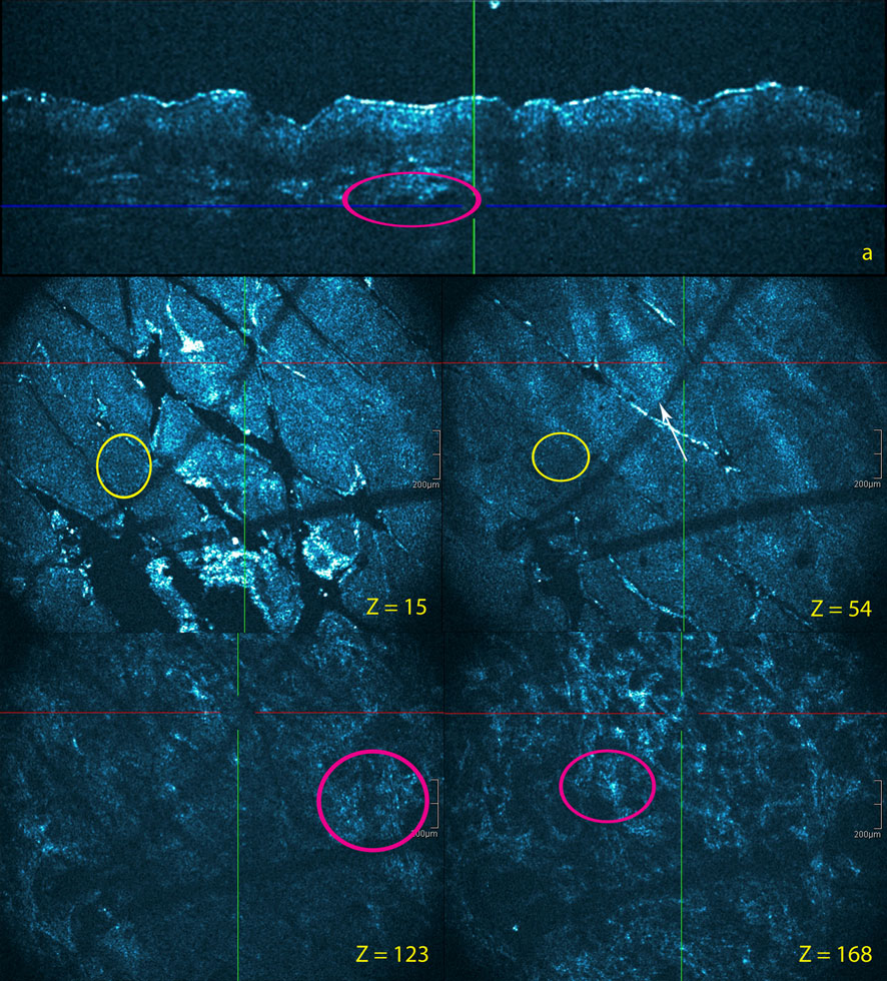
Subacute lesion in atopic dermatitis patient. Cross-sectional (**a**) and en face HD-OCT images (*z* values indicated). No significant hyperkeratosis is observed. Because of spongiosis (*yellow circles*), a small increase of epidermal thickness compared to the normal skin can be observed. Exocytosis (*white arrow*) of inflammatory cells can be demonstrated epidermal acanthosis is minimal with some elongation of rete ridges. The papillary dermal edema is minimal. There is no papillary dermal thickening. There is a clear dermal perivascular inflammation (*violet circles*)

**Fig. 3:**
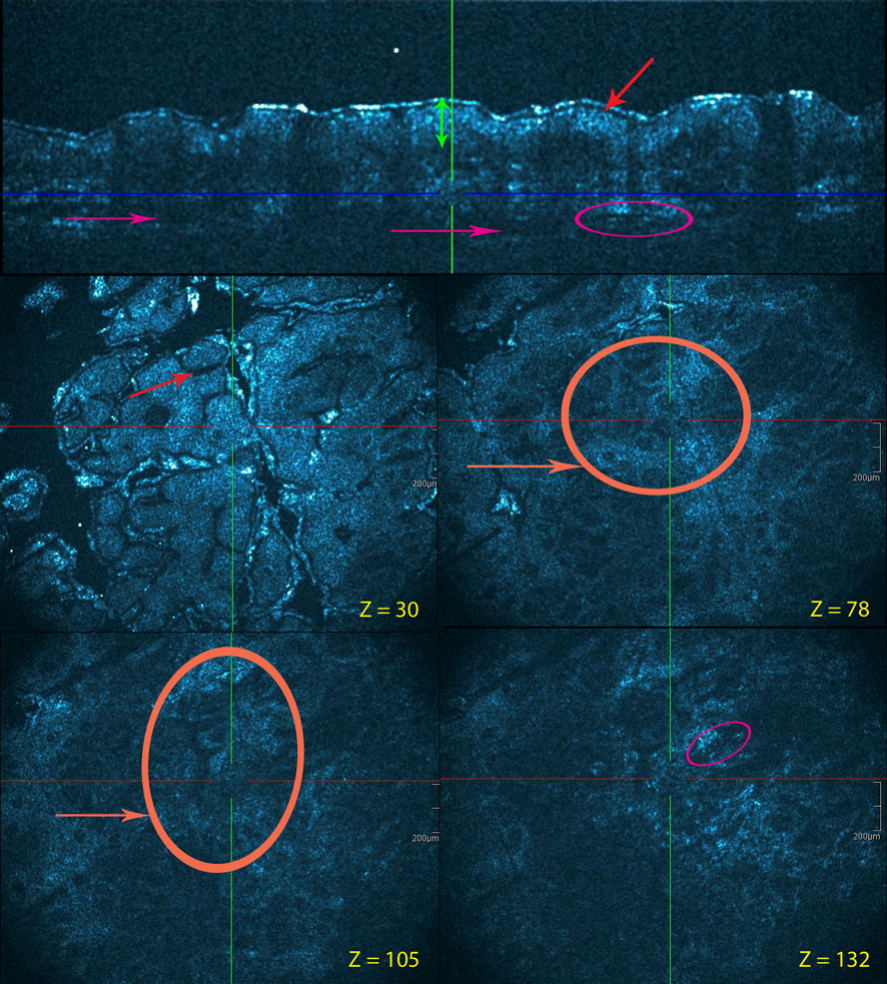
Chronic lesion in atopic dermatitis patient. Cross-sectional and en face HD-OCT images (*z* values indicated). Hyperkeratotic orthokeratosis is noticed (*red arrows*). Moderate epidermal acanthosis (*green double arrow*). Spongiosis is minimal and no intraepidermal microvesicles are observed. The papillary dermal thickening is pronounced with papillomatosis (*orange circle and arrow*). There is vascular dilatation (*violet arrows*) and dermal perivascular inflammation (*violet circle*)

HD-OCT imaging of these skin conditions clearly showed the presence of spongiosis or intercellular edema that stretches apart keratinocytes and sometimes results in the formation of intraepidermal vesicles. Sometimes exocytosis of inflammatory cells could be identified. Morphologic features correlating with the acute, subacute and chronic stages could be observed (Table 3). In *the acute form* (Fig. 1), there was no hyperkeratosis. Compared to normal skin [12, 13, 18, 24], a significant increase (>50 %) in epidermal thickness was observed due to spongiosis. Intercellular edema decreased the reflectivity from cellular layers. Intraepidermal microvesicles were present, sometimes with macrovesicles in case of severe allergic reactions. Migration of inflammatory cells into the epidermis (exocytosis) was presented. There was no epidermal hyperplasia (acanthosis). In moderate allergic reactions, no dermal papillary thickening was observed. Inflammatory edema in the dermis appeared to lower dermal reflectivity. In moderate forms, dilated signal-free cavities in the dermis corresponding to blood vessels and lymphatic vessels were noticed. In these forms, the dermal perivascular inflammation was marked with highly reflective inflammatory cells. In *the subacute form* (Fig. 2), no significant hyperkeratosis was observed. Small mounds of parakeratosis occured in the keratin layer over the spongiotic vesicles. Because of spongiosis, a moderate increase (±10 %) of epidermal thickness compared to the normal skin [12, 13, 18, 24] could be observed. Spongiotic vesicles were seen. Acanthosis was minimal with some elongation of rete ridges. The papillary dermal edema was minimal. There was no papillary dermal thickening. There was a clear dermal perivascular inflammation. In *chronic spongiotic dermatitis* (Fig. 3), hyperkeratotic orthokeratosis was noticed in which localized patches of parakeratosis could be observed. There was also localized patchy hypergranulosis (Table 3). Moderate (±10 %) to marked (±30 %) epidermal acanthosis was also observed. Spongiosis was minimal to absent and no intraepidermal microvessel was observed. The papillary dermal thickening was pronounced with papillomatosis. There was dermal perivascular inflammation.

#### Psoriasiform dermatitis

**Fig. 4:**
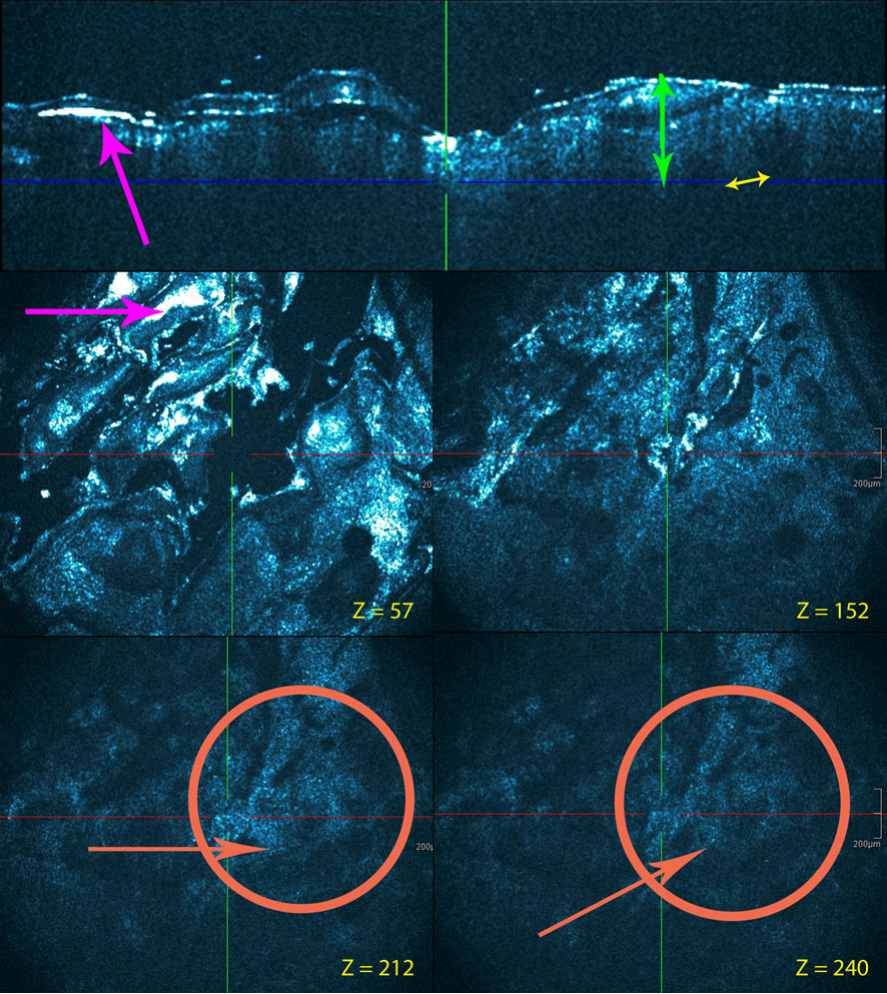
Leg, chronic plaque psoriasis. Cross-sectional and en face HD-OCT images (*z* values indicated). Hyperkeratosis is pronounced. Refractile structures in the stratum corneum correspond to parakeratosis (*violet arrows*). Acanthosis with a more regular elongation and broadening of the rete ridges is presented (*green double arrow*). Between these enlarged rete ridges (*yellow double arrow*), the dermal papillae are swollen by edema. Papillary rings are less brighter than normal. The dermo-epidermal junction is also less defined due to psoriasiform papillomatosis with an increased number and density of papillary rings (*orange circle and arrow*)

**Fig. 5:**
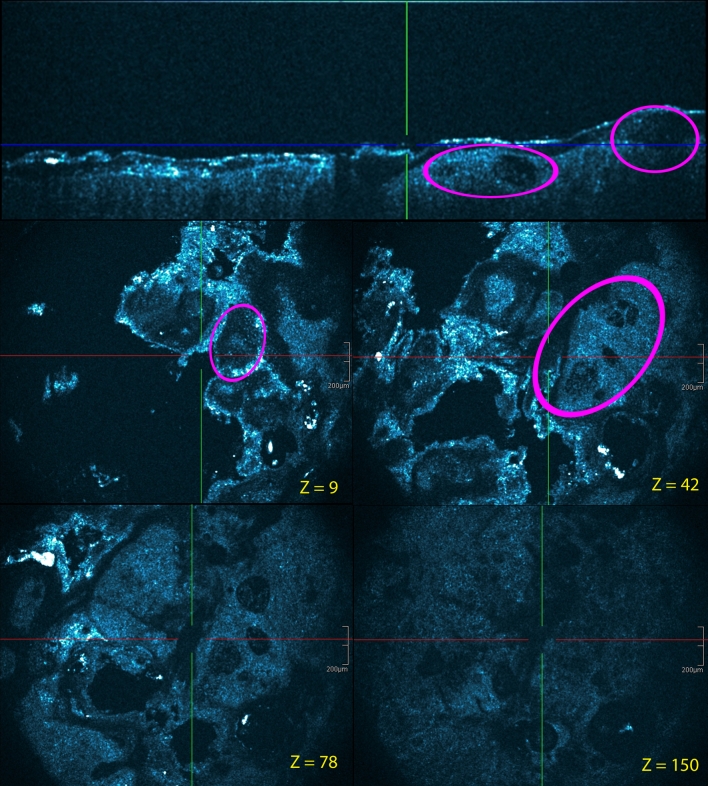
Chronic palmar psoriasis lesion. Cross-sectional and en face HD-OCT images. Collections of neutrophils are found in the stratum corneum (*pink circles*). These lesions correspond to Munro microabscesses. *z* values are indicated

Using chronic plaque psoriasis as the morphological prototype, a number of features could be observed in HD-OCT images (Fig. 4). Hyperkeratosis was pronounced. Hyper-reflective structures in the stratum corneum were corresponding to parakeratosis. Absence or reduction of the granular cell layer was regularly observed. The honeycomb pattern of the epidermis was conserved, but acanthosis with a more regular elongation and broadening of the rete ridges was present. At the top of the dermal papillae, a thinner epidermis was frequently noticed. Between the enlarged rete ridges, the dermal papillae were swollen due to edema and contained prominent dilated capillaries. A disappearing of the brighter papillary rings was often observed. The more pronounced the inflammation, the more dim the papillary rings became. The dermo-epidermal junction was also less well defined due to psoriasiform papillomatosis with an increased number and density of papillary rings. The reflectivity in the papillary dermis was lower than that in healthy skin suggesting an important papillary edema. Dilated blood vessels are visible surrounded by an important perivascular inflammatory infiltrate. Munro’s micro abscesses could be demonstrated in two patients (Fig. 5).

#### Interface dermatitis

**Fig. 6:**
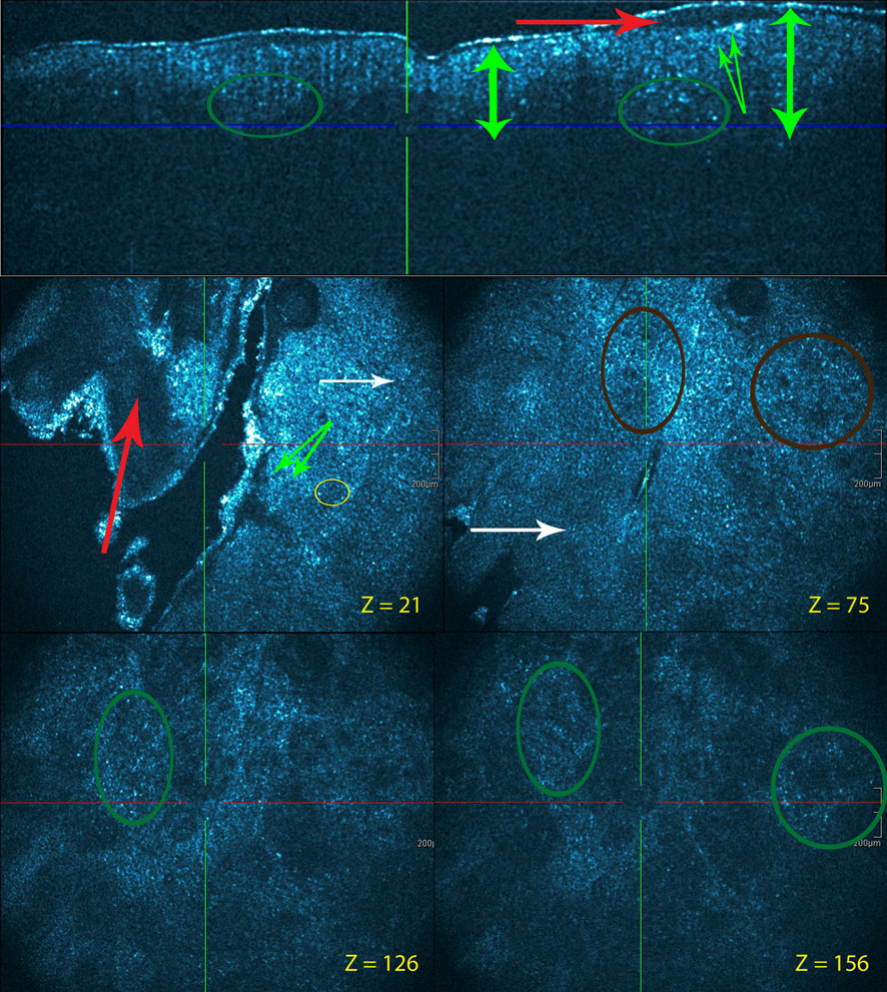
Lichen planus: lesion on the calf. Cross-sectional and en face HD-OCT images. Hyperkeratosis (*red arrows*) is present without parakeratosis. Irregular thickening of granular layer (*light*-*green arrow*s) and acanthosis (*green double arrow*s), with irregular lengthening of rete ridge is observed. Moderate spongiosis can be observed (*yellow circle*). Degeneration of epidermal basal layer with necrotic keratinocytes visualized as total obliteration of the ring-like structures around the dermal papillae. Heavy dermal inflammatory cell infiltrate is diffuse and closely applied to dermal-epidermal junction (*dark*-*green circles*). Inflammatory cells are also present in the epidermis (*white arrow*s). The combination of irregular lengthening of rete ridges, basal layer destruction and upper dermal edema and inflammatory infiltrate, produces a “saw-tooth” appearance of rete ridges. *z* values are indicated

A consistent HD-OCT feature of Lichen planus (Fig. 6) as a prototype of lichenoid-interface dermatitis appeared to be hyperkeratosis coupled with rare or absent parakeratosis. Irregular thickening of granular layer and acanthosis, with irregular lengthening of rete ridge was observed. Moderate spongiosis could be observed. Degeneration of epidermal basal layer with necrotic keratinocytes visualized as total obliteration of the ring-like structures around the dermal papillae. Dermal inflammatory cell infiltrate, usually heavy, was diffuse and closely associated to the DEJ. Inflammatory cells were also present in the epidermis. Sparse dilated vessels and sparse dermal sclerosis were seen. The infundibula were normal.

**Fig. 7:**
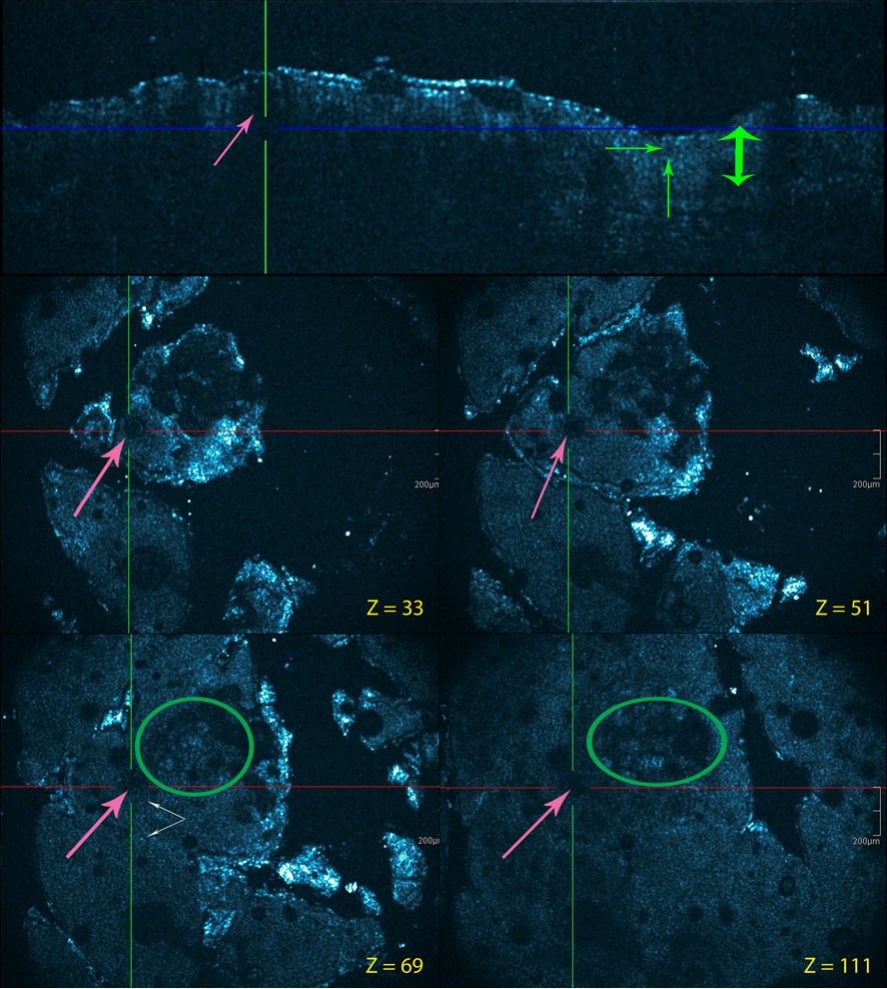
Discoid lupus erythematosus: facial lesion. Cross-sectional and en face HD-OCT images. Dilated hyperkeratotic infundibula (*light*-*magenta arrow*s). Irregular thickening of the epidermis with hypergranulosis (*light*-*green arrow*s) and acanthosis (*green double arrow*). Necrotic keratinocytes and inflammatory cells (*white arrows*) are present in the epidermis. Interface changes with high reflective cells at the level of the dermo-epidermal junction as singles and clusters (*dark*-*green circles*). This is associated with a partial obliteration of ring-like structures around the dermal papillae. *Z* values are indicated

In contrast, HD-OCT features of discoid lupus erythematosus (Fig. 7) as a prototype of vacuolar-interface dermatitis without ballooning showed dilated hyperkeratotic infundibula. Interface changes appeared here with high reflective cells at the level of the dermo-epidermal junction in singles or as clusters. This was associated with a partial (focal) obliteration of ring-like structures around the dermal papillae. Irregular thickening of the epidermis with hypergranulosis and acanthosis could be seen, and necrotic keratinocytes were present. Inflammatory cells were also present in the epidermis. There was a clear dermal inflammation with dilated vessels. In more chronic forms, the epidermal was more atrophic together with a thickened basal membrane area.

#### Erythema multiforme as prototype of ballooning dermatitis

**Fig. 8:**
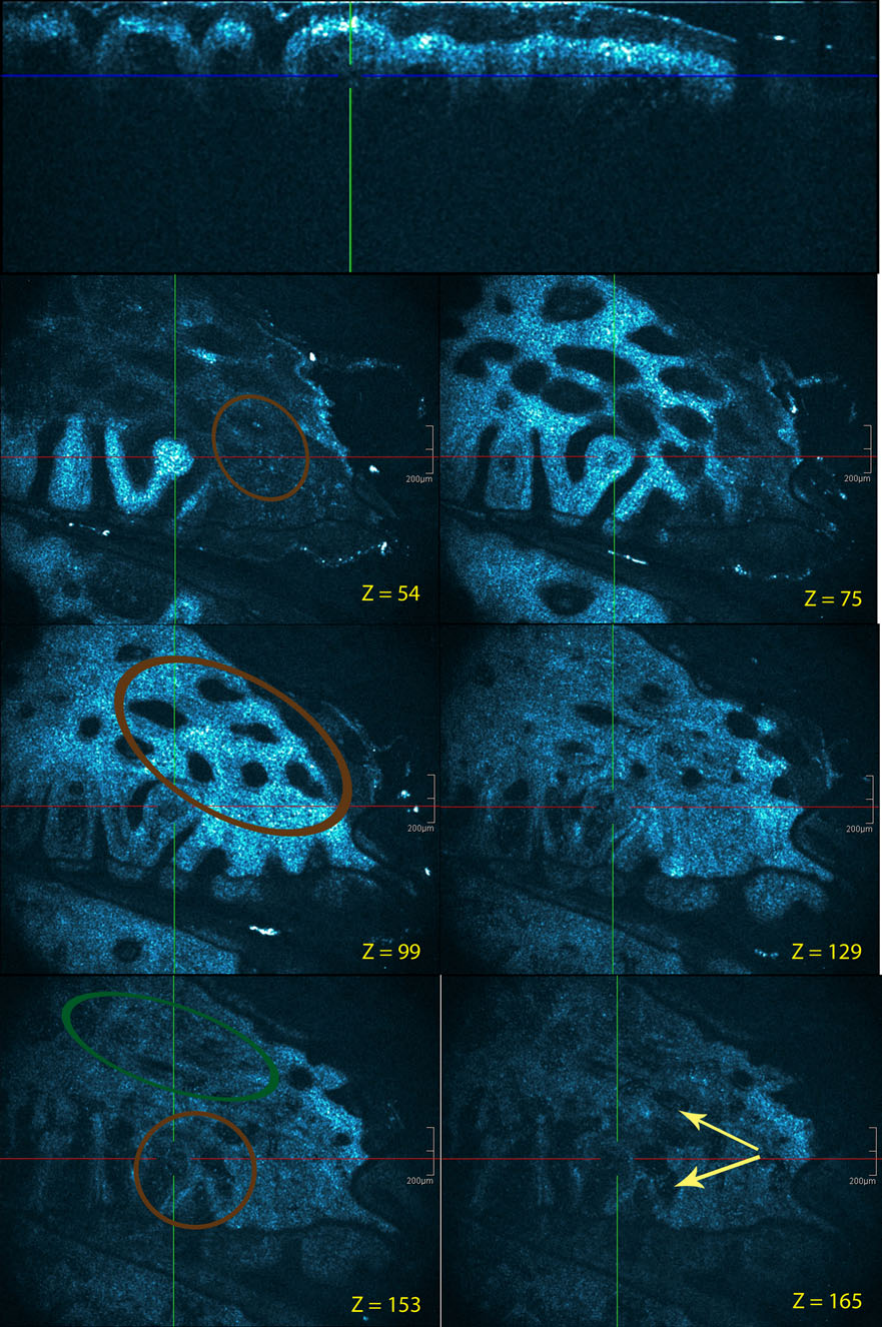
Drug-induced erythema multiforme: palm. Cross-sectional and en face HD-OCT images (*z* values indicated). Necrotic keratinocytes were generally present at all epidermal levels (*brown encircled*) (*z* = 54). Ballooning degeneration of spinous cells (*brown circle*) (*z* = 99). At the junction between the epidermis and dermis, small, discrete vacuoles are observed (*brown circle*) (*z* = 153). Partial obliteration of the ring-like structures around the dermal papillae (*dark*-*green circle*). Melanophages can also be observed in papillary dermis (*light*-*yellow arrow*s)

HD-OCT features of ballooning dermatitis (Fig. 8) as seen in drug-induced erythema multiforme included variable degrees of epidermal necrosis. Necrotic keratinocytes were present at all epidermal levels. Ballooning of spinous cells was also seen with vacuolar alteration of the basilar epidermis (basal vacuolization). At the junction between the epidermis and dermis, small, discrete vacuoles and characteristic melanophages were seen. Papillary dermis was edematous with dilated capillaries. There was a perivascular infiltrate.

### An algorithm based on pattern analysis at intermediate (10×) magnification (Fig. 9)

*Step one*: Determination of the basic pattern formed by infiltrates of cells. The perivascular dermatitis by far the most common group was selected for this study. The other patterns defined by Ackerman et al. [1] have been excluded. The inflammatory infiltrate around venules of the superficial plexus in the papillary dermis and around venules of the upper part of the reticular dermis could be observed. An infiltrate deeper than 570 μm could not be detected by the HD-OCT.

**Fig. 9:**
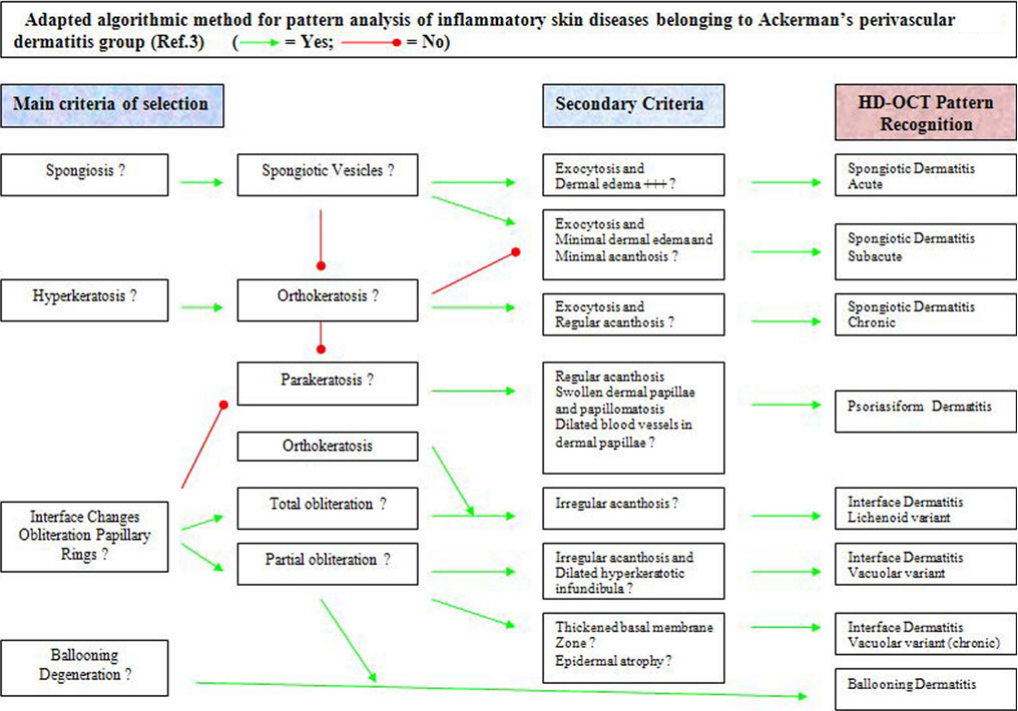
Adapted algorithmic method for pattern analysis of inflammatory skin diseases belonging to Ackerman’s perivascular dermatitis group [3]

*Step two*: Division of the perivascular dermatitis pattern: This group can be separated into perivascular alone with no apparent epidermal involvement or perivascular with involvement of the epidermis. This study has been focused only on the perivascular dermatitis group with apparent epidermal involvement. Four main criteria have been found leading to specific subgroup diagnosis: A: spongiosis with or without spongiotic vesicles, B: orthokeratosis or parakeratosis, C: interface changes with partial or total obliteration of papillary rings and D: ballooning degeneration. The secondary criteria of selection were: pronounced dermal edema, exocytosis, regular or irregular acanthosis, papillomatosis, swollen dermal papillae, dilated blood vessels in dermal papillae, epidermal atrophy, thickened basal membrane area and dilated hyperkeratotic infundibula.

The combination of main criteria and secondary criteria could lead to a more specific subgroup diagnosis (Fig. 9). Acute spongiotic dermatitis is characterized by a combination of spongiosis with spongiotic vesicles, exocytosis and dermal edema. The chronic form presented a mixture of orthokeratosis, spongiosis without spongiotic vesicles, exocytosis and regular acanthosis. Psoriasiform dermatitis is characterized by a combination of parakeratosis, regular acanthosis, no obliteration but hyporeflectivity of papillary rings, papillomatosis, swollen dermal papillae and dilated blood vessels in dermal papillae. The lichenoid variant of interface dermatitis presented orthohyperkeratosis, irregular acanthosis and total (diffuse) obliteration of papillary rings. The vacuolar variant of interface dermatitis in this study was discoid lupus erythematosus and presented irregular acanthosis, partial (focal) obliteration of papillary rings and dilated hyperkeratotic infundibula. In more chronic forms, the epidermal atrophy was pronounced together with a thickened basal membrane area. A special variant of the perivascular dermatitis group is the ballooning dermatitis and was characterized by necrotic keratinocytes and ballooned spinous cells in combination with a focal obliteration of papillary rings.

*Step three*: Identification of the inflammatory cells that make up the infiltrate such as lymphocytes and granulocytes. Specific types of inflammatory cells often can be recognized for what they are as follows: lymphocytes (diameter 7–8 μm) are high reflective small dots that seem to be about equidistant from one another because they have a solitary uniform nucleus. Neutrophils (diameter 10–12 μm) having a smaller multilobed nucleus are somewhat less reflective but larger dots (Fig. 5).

## Discussion

Skin imaging potentially allows much more than the possibility of non-invasive diagnosis in a clinical setting. It may also provide a dynamic method for the study of how lesions evolve and thereby contribute to our basic understanding of skin biology. One important approach to our understanding of lesional evolution is, fortunately, through diagnostic studies. As stated, two main options exist for in vivo imaging: either to develop a method offering sufficient optical resolution or to establish reliable pattern recognition at a lower magnification.

Over the years, a series of imaging techniques have been developed with varying degrees of resolution and penetration. Currently, RCM has the best resolution, albeit at the cost of a very limited depth of imaging. In consequence, RCM criteria for a number of diagnoses have been established comparing the method with histology [2–6, 14–17, 19, 20, 22, 23, 25, 26]. HD-OCT is a promising high-resolution imaging technique which offers comparable resolution to RCM and improved penetration [9–11]. This study suggests that HD-OCT features of inflammatory skin conditions with epidermal alteration correlate well with dermatopathologic descriptors as defined in RCM. The morphological features imaged by HD-OCT were very similar to those described for RCM. Furthermore, it was possible to apply pattern recognition derived from intermediate magnification histopathology meaningfully to the HD-OCT images.

HD-OCT allowed visualization of cellular infiltrates, although identification of the inflammatory cells that make up the infiltrate such as lymphocytes and granulocytes remains difficult for both RCM and HD-OCT. Cytological changes such as ballooning are however easily seen. One of the restrictions of RCM in evaluating inflammatory skin diseases is the limited penetration depth [2–6, 14–17, 19, 20, 22, 23, 25, 26]. With HD-OCT not only the visualization and assessment of individual cells but also microanatomic structures can be performed to a depth of 570 μm. This allows the method to identify specific patterns such as spongiosis, interface dermatitis and acanthosis. The method therefore appears to be able to identify the components needed for pattern analysis.

Images of common inflammatory skin diseases were therefore evaluated by applying Ackerman’s algorithmic method of pattern recognition at intermediate (10×) magnification. Of Ackerman’s eight essential patterns of inflammatory skin diseases, perivascular dermatitis is the most common by far. Most of the common inflammatory skin diseases are perivascular dermatitides among those being allergic contact dermatitis, lichen planus and psoriasis. A perivascular dermatitis is identified by the presence of inflammatory cells around venules situated either in the upper part of the reticular dermis (superficial plexus) or in both the upper and lower parts of the reticular dermis (superficial and deep vascular plexuses) [1].

Once a pattern of perivascular dermatitis has been identified, it is necessary to make an evaluation about whether the epidermis, including the dermo-epidermal junction, is involved in the process. If the epidermis is affected, the pattern may be one in which spongiosis is discernible (spongiotic dermatitis), or the dermo-epidermal interface is obscured (interface dermatitis) or spinous cells are ballooned (ballooning dermatitis) or psoriasiform acanthosis is apparent (psoriasiform dermatitis). Just like RCM, HD-OCT allows discriminating between these inflammatory reaction patterns, the only difference appears to be the increased depth of the tissue that can be visualized by HD-OCT.

Acute allergic spongiotic dermatitis is characterized by spongiosis, spongiotic vesicles and dermal edema in both slice and en face HD-OCT imaging. Hyperkeratosis without parakeratosis, acanthosis and the presence of papillary rings are typical features for chronic spongiotic dermatitis. Chronic plaque psoriasis is characterized by hyperkeratosis with parakeratosis, regular acanthosis, papillomatosis with swollen, edematous dermal papillae and dilated blood vessels in slice and en face HD-OCT imaging. An important finding for chronic plaque psoriasis is the disappearing of the brighter papillary rings especially when the inflammation was pronounced. The decreased melanin content of the keratinocytes of the basal cell layer is most probably responsible for this observation. α-MSH is a well-known mediator of skin pigmentation. More recently, it has been shown that α-MSH also exerts a strong anti-inflammatory and immunosuppressive activities [7]. The observed attenuation of the brightness of the papillary rings could be a morphologic correlate of the neuro-immunological abnormalities in chronic psoriatic lesions. HD-OCT features of interface changes are the presence of small reflective cells at the level of the dermo-epidermal junction in singles or clusters. This is also associated with total or partial obliteration of the ring-like structures around the dermal papillae. In ballooning dermatitis, such as erythema multiforme ballooning, degeneration of cells of the stratum spinosum is observed combined with focal interface changes. Typical HD-OCT features of discoid lupus erythematosus as prototype of vacuolar-interface dermatitis without ballooning are the focal interface changes and the dilated hyperkeratotic infundibula. Hyperkeratosis without parakeratosis, irregular acanthosis and almost total obliteration of papillary rings are strong HD-OCT descriptors for Lichen planus as prototype of lichenoid-interface dermatitis.

Based on these findings, a HD-OCT-adapted algorithmic method for pattern analysis of common inflammatory skin diseases belonging to Ackerman’s perivasculitis group [1] is proposed in Fig. 9.

Studies are currently undertaken to evaluate the possibilities and limitations of HD-OCT imaging in Ackerman’s seven other essential patterns of inflammatory skin diseases.

Histopathological assessment of inflammatory skin diseases remains the gold standard of diagnosis of inflammatory skin diseases. Although the discussed inflammatory skin diseases have more characteristic clinical findings, making utility of in vivo non-invasive imaging techniques limited, HD-OCT represents an interesting tool for rapid imaging of these skin diseases. Despite the above-mentioned limitations, HD-OCT imaging offers a unique opportunity to analyze, to guide the best location of a biopsy and to monitor over time inflammatory skin diseases non-invasively at a cellular resolution. Blind evaluations and inter-observer reproducibility are mandatory. Therefore, additional studies to test the sensitivity and specificity of the proposed algorithm for pattern analysis are essential to validate the findings of this pilot study.
